# Association of Ambulance Use in New York City With the Implementation of the Patient Protection and Affordable Care Act

**DOI:** 10.1001/jamanetworkopen.2019.6419

**Published:** 2019-06-28

**Authors:** Charles Courtemanche, Andrew I. Friedson, Daniel I. Rees

**Affiliations:** 1 Department of Economics, Gatton College of Business and Economics, University of Kentucky, Lexington; 2 IZA Institute of Labor Economics, Bonn, Germany; 3 National Bureau of Economic Research, Cambridge, Massachusetts; 4 Department of Economics, University of Colorado Denver, Denver

## Abstract

**Importance:**

Expanding insurance coverage may be associated with overuse of medical care because newly insured patients are insulated from having to pay the full cost.

**Objective:**

To examine the use of ambulance transport before and after the rollout of the Patient Protection and Affordable Care Act (ACA) in New York City (NYC), New York.

**Design, Setting, and Participants:**

In this case-control study, the volume of ambulance dispatches in NYC for minor injuries before and after the rollout of the ACA was examined. Data were drawn from a census of all ambulance dispatches in NYC between January 1, 2013, and July 31, 2016. Ambulance dispatches for more severe injuries, which are more difficult to characterize as unnecessary, were used as the control group. Analyses were conducted from August 17, 2017, to May, 10, 2019.

**Main Outcome and Measures:**

The main outcome was the number of ambulance dispatches for minor injuries, defined per month per dispatch zone. The implementation of the ACA was measured using an indicator variable of 1 for dispatches starting January 1, 2014, and 0 for dispatches before January 1, 2014. The number of ambulance dispatches for injuries and major injuries was used to account for secular trends. Injury severity was classified by the dispatchers based on information from the 911 callers with a severity score on a scale of 1 to 8, where 1 is the most severe; minor injuries had a score of 7; injuries, 5; and major injuries, 3.

**Results:**

There were 4 787 180 ambulance dispatches in NYC during the study. After the 2014 expansion of insurance coverage under the ACA, there was an increase in ambulance dispatches for minor injuries compared with dispatches for more severe injuries. Compared with the preimplementation mean (SD) of 20.75 (14.24) minor injury dispatches per dispatch zone per month, there were 7.71 (95% CI, 1.23-14.19) additional minor injury dispatches per dispatch zone per month compared with dispatches for other types of injuries, an increase of 37.2%. Given that NYC has 31 dispatch zones, this increase is equivalent to approximately 239 additional dispatches per month or 2868 additional dispatches per year for minor injuries.

**Conclusions and Relevance:**

There was a significant increase in use of ambulance transport for minor injuries in NYC after the ACA insurance expansion, suggesting that the change in health insurance structure was associated with increased use of emergency medical services in nonemergent situations. Future reforms to the US health insurance system should take into account the potential for increased use of emergency medical services in nonemergent situations, which the literature suggests may lead to congestion and slower response times.

## Key Points

**Question:**

Was the expansion of insurance coverage under the Patient Protection and Affordable Care Act associated with changes in ambulance service use in New York, New York?

**Findings:**

In this case-control study of more than 4.7 million ambulance transports in New York City, from January 1, 2013, to July 31, 2016, the expansion of insurance implemented under the Patient Protect and Affordable Care Act was associated with a statistically significant increase in ambulance dispatches for minor injuries compared with ambulance dispatches for more severe injuries.

**Meaning:**

Insurance expansion may be associated with increased use of emergency medical services in nonemergent situations, which the literature suggests may lead to congestion and slower response times.

## Teaser

This case-control study of emergency medical service dispatch data in New York, New York, examines changes in dispatches of ambulance transports for minor injuries compared with other injuries before and after the implementation of the Patient Protection and Affordable Care Act.

## Introduction

There is evidence dating back to the 1987 RAND Health Insurance Experiment^1^ that expanding health insurance coverage leads to increased health care utilization. For example, shortly after qualifying for Medicare, individuals are more likely to be admitted to the hospital and more likely to have elective procedures.^2^ Researchers have also documented increased health care utilization after state Medicaid expansions, the 2006 Massachusetts universal coverage initiative, the 2010 dependent coverage expansion under the Patient Protection and Affordable Care Act (ACA), and the 2014 expansions of private and public coverage under the ACA.^3,4,5,6,7^

These findings are relevant to policy makers and health care experts, but there is also interest in whether, by insulating patients from having to pay full cost, insurance coverage expansions lead to the use of medically unnecessary care. One potentially fruitful approach to testing whether insurance coverage expansions are associated with the use of medically unnecessary care is to focus on emergency medical services (EMS) use. In the presence of limited resources for health care, an increase in the use of EMS could lead to congestion, slower overall EMS response times, and even deaths.^8,9^

Our study examined the volume of EMS dispatches in New York City (NYC), New York, before and after the implementation of ACA provisions that expanded insurance coverage and generosity through a combination of insurance market reforms, mandates, subsidies, and Medicaid expansions. These provisions took effect in 2014^6,10,11,12,13^ and included the rollout of essential health benefits, which required all insurance plans to cover ambulance transport (among other services). Even if many NYC residents did not experience a change in their health insurance coverage status with the rollout of the ACA, they could have experienced an increase in coverage generosity, which in turn could have increased their demand for EMS.

Using a census of all EMS dispatches in NYC, in addition to the dispatcher’s best assessment of the patient’s diagnosis (which was based on all of the available information at the time of the ambulance dispatch), our study explored how the composition of EMS dispatches changed after the 2014 implementation of the ACA. Specifically, the objective was to determine whether there was an increase in ambulance transports for minor injuries after implementation compared with ambulance transports for injuries and major injuries.

## Methods

Data from the NYC EMS Incident Dispatch Data (EIDD) file from January 1, 2013, through July 31, 2016, were analyzed. Analyses were conducted from August 17, 2017, to May, 10, 2019. The EIDD file contains deidentified information on all ambulance dispatches in NYC and is available free of charge through NYC Open Data.^14^ Under Local Law 11 of 2012, all public data collected by NYC must be made available through a single web portal.^14^ Analyses of secondary, deidentified data are considered exempt from requiring institutional review board approval by the University of Colorado Institutional Review Board. This study followed the Strengthening the Reporting of Observational Studies in Epidemiology (STROBE) reporting guideline.

New York City uses a central 911 dispatch for all emergency calls that then routes medical emergencies to trained EMS dispatchers.^15^ In addition to time and location, the EIDD file includes the initial call code for each dispatch. When someone calls 911 and reaches an EMS dispatcher in NYC, the dispatcher asks a series of questions to triage the call based on type and severity. The answers to these questions are put into a computer algorithm (ie, a decision tree) that assigns a call code and associated severity score. The eTable in the Supplement presents the most common call codes and their associated severity levels. Although the call code and severity score do not amount to a diagnosis and do not necessarily reflect the actual condition of the patient, they can be thought of as providing a reasonably accurate assessment based on all of the available information immediately prior to the dispatch of the ambulance. Aside from the call code and severity score, no other information about the patient is available in the EIDD file.

There are 31 dispatch zones in NYC, each served by multiple ambulances at any given time. Ambulances can be owned and operated by private companies, but every ambulance is dispatched centrally, and all ambulance dispatches, regardless of ownership, appear in the EIDD file.^16^ Dispatch data after July 31, 2016, were not available from the EIDD file at the time of our analysis, so the focus of this study was on the short-term associations of expanding insurance coverage with ambulance dispatches. When data from after July 31, 2016, become available, it will be possible to gauge the longer-term association of expanding insurance coverage with ambulance dispatches in NYC.

Ambulances dispatches in NYC are tracked via electronic computer records (as opposed to using paper records), and the EIDD file contains detailed information about each incident, including the dispatch time and location information. This information was aggregated to the zip code level before being made public to comply with patient privacy protections. Therefore, there is no information of any kind on demographic characteristics included in the EIDD file. Dispatch counts from the EIDD file were aggregated to the dispatch zone on a monthly level for this analysis.

Two types of EMS dispatches, which are mutually exclusive, were examined. The first type is composed of dispatches with a call code of minor injury. Dispatches for minor injuries receive a severity score of 7 on a scale of 1 to 8, in which 1 is the most severe score. Examples of minor injuries include shallow cuts or abrasions, minor burns covering only a small area of skin, and muscle sprains.

The second type of EMS dispatch is composed of dispatches with a call code of injury or major injury. These dispatches have severity scores of 5 for injury and 3 for major injury. Examples of nonminor injuries include compound fractures; injuries accompanied by chest pain, paralysis, confusion, severe bleeding, or unconsciousness; and any type of head or eye injury.

The volume of EMS injury dispatches is a function of many factors, such as population changes, changing demographic characteristics, and fluctuations in economic activity.^17^ By comparing EMS dispatches for minor injuries with dispatches for more severe injuries, any underlying common secular trends common to all types of injuries are controlled for via the difference-in-differences method. The outcome for this analysis was the number of EMS dispatches of a given type (ie, minor injuries vs injuries and major injuries) in a given month and ambulance dispatch zone.

### Statistical Analysis

A difference-in-differences analysis was conducted. The goal of this analysis was to estimate the association of ACA implementation with EMS dispatches for minor injuries vs dispatches for all other types of injuries.^18^ A linear regression model was fitted using the ordinary least squares method: *λ_czmy_ = β_0_ + β_1_(Minor_c_ × Post_y_) + β_2_Minor_c_ + β_3_Post_y_ + β_4_Pop_zy_ + θ_z_ + θ_m_ + ε_czmy_*, where *λ_czmy_* is the count of dispatches of type *c* in dispatch zone *z*, month *m*, and year *y*. Because there were 2 possible call types, 31 dispatch zones in NYC, and 42 months of data, the regression was based on 2604 observations (2 × 31 × 42 = 2604). The variable *Minor_c_* is coded as 1 for dispatch counts for minor injuries and 0 for dispatch counts for all other types of injuries. *Post_y_* indicates whether the ACA had been implemented by year *y*. *Post_y_* was coded as 1 as of January 1, 2014, and 0 before that date. The coefficient for the interaction of *Minor*_c_ with *Post_y_*, *β_1_*, is the difference-in-differences estimator, which represents the change in minor injury dispatches after the rollout of the ACA compared with dispatches for other types of injuries. In other words, the association of the ACA with dispatches for minor injuries was estimated using dispatches for nonminor injuries as the control group. *Pop_zy_* is the population of dispatch zone *z* in year *y* and was constructed using information from the 2013 to 2017 American Community Survey.^19^ Dispatch zone fixed effects are represented by *θ_z_*, and month fixed effects are represented by *θ_m_*. To account for the count nature of the outcome variable, the association of the implementation of the ACA with minor injury dispatches was also estimated using negative binomial regression analysis.

In addition to providing standard difference-in-differences estimates based on the interaction of *Minor_c_* with *Post_y_*, the variable *Minor_c_* was interacted with a series of indicators, each of which corresponded to a different quarter (ie, a 3-month period) in the data. There were 14 quarters in the data, but the first quarter indicator (January 1 to March 31) of 2013 was omitted to provide a baseline.

The rationale for plotting the estimated coefficients of these interactions is 2-fold. First, by plotting them, it is possible to ascertain whether dispatch counts for minor injuries and other types of injuries were on different trajectories before the ACA rollout in 2014. Statistically significant differences in prereform trends (but not levels) would violate the parallel trends assumption. The difference-in-differences estimator, β_1_, is only valid when this assumption holds.

Second, plotting these estimated coefficients allows for the construction of an event-study figure and an in-depth exploration of post-ACA ambulance dispatch dynamics. For example, it is possible that the post-ACA dispatch trends by type gradually diverged over time. Although the gradual divergence in post-ACA trends would be not be captured by a standard difference-in-differences estimate, it would be readily apparent from an event-study figure.

For all regression estimates, 95% CIs were constructed using cluster-robust standard errors at the call type–dispatch zone level.^20^ Two-sided hypothesis tests were used to determine statistical significance. Statistical significance was set at *P* less than .05. Data were analyzed using Stata version 14.2 (StataCorp).

## Results

There were 4 787 180 EMS dispatches in NYC during the study. Dispatches for minor injuries represented less than 1% of total EMS dispatches in NYC during the study. Dispatches for injuries and major injuries represented 16% of total EMS dispatches in NYC during the study. The mean (SD) minor injury dispatches per zone per month prior to the implementation of the ACA was 20.75 (14.24). After December 31, 2013, the mean (SD) dispatches for minor injuries per zone per month was 33.14 (23.72), an increase of 62.7% (Table 1). By contrast, dispatches for other types of injuries held reasonably steady during the study. There was a mean (SD) of 555.54 (299.86) dispatches for other types of injuries per dispatch zone per month prior to the implementation of the ACA in 2014. In the post-ACA period, there was a mean (SD) of 560.21 (299.67) dispatches for other types of injuries per dispatch zone per month, an increase of 0.8% (Table 1).

**Table 1.** Summary Statistics for Ambulance Dispatch Counts in New York City, New York, From January 1, 2013, to July 31, 2016^a^

Year	Monthly Dispatches per Dispatch Zone, Mean (SD), No.	
	Minor Injury	Other Injury
2013	20.75 (14.24)	555.54 (299.85)
2014	25.41 (17.15)	559.16 (298.87)
2015	38.76 (26.90)	563.44 (303.97)
2016	37.33 (24.19)	555.83 (294.07)

^a^ Injury definitions are based on the 911 emergency medical services dispatcher’s conversation with the caller and represent the dispatcher’s best assessment of the patient’s status. Dispatches for minor injuries receive a severity score of 7 on a scale of 1 to 8, in which 1 is the most severe score. Examples of minor injuries include shallow cuts or abrasions, minor burns covering only a small area of skin, and muscle sprains. Dispatches for other types of injuries have severity scores of 5 (injury) and 3 (major injury). Examples of other, nonminor injuries include compound fractures; injuries accompanied by chest pain, paralysis, confusion, severe bleeding, or unconsciousness; and any type of head or eye injury.

**Figure 1.**
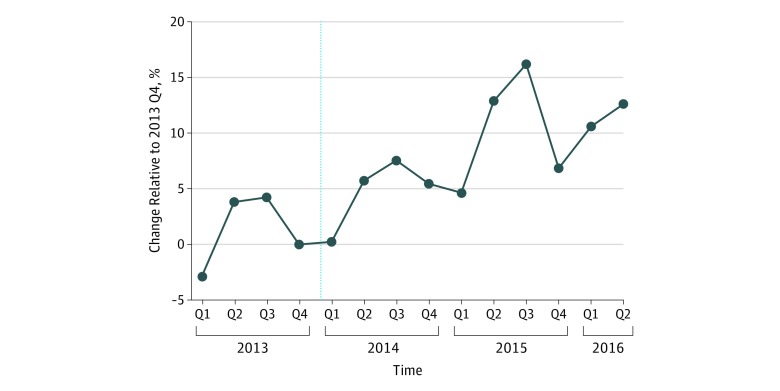
Total Ambulance Dispatches in New York City, New York, From January 1, 2013, to July 31, 2016 Dispatch volume is reported as a percentage change compared with the fourth quarter (Q) of 2013. There were a total of 321 296 dispatches in 2013 Q4. The dashed line indicates the implementation of the Patient Protection and Affordable Care Act.

Figure 1 presents percentage changes in the volume of all EMS dispatches in NYC from January 1, 2013, to July 31, 2016, compared with the baseline final quarter of 2013, when 321 296 ambulances were dispatched in NYC. The last quarter before the implementation of the ACA increased insurance coverage (owing to the opening of the ACA exchanges and Medicaid expansion) and mandated that emergency services, including ambulance rides, be covered by all health plans as an essential health benefit.

The volume of ambulance dispatches increased steadily after the implementation of the ACA, with some seasonal variation. The volume of EMS dispatches reached a high of 377 720 in the third quarter of 2015, or an 18% increase compared with baseline. It is difficult to ascertain how much, if any, of this increase in total EMS dispatches was associated with the ACA and how much was associated with other factors, such as population increases and economic activity.

**Figure 2.**
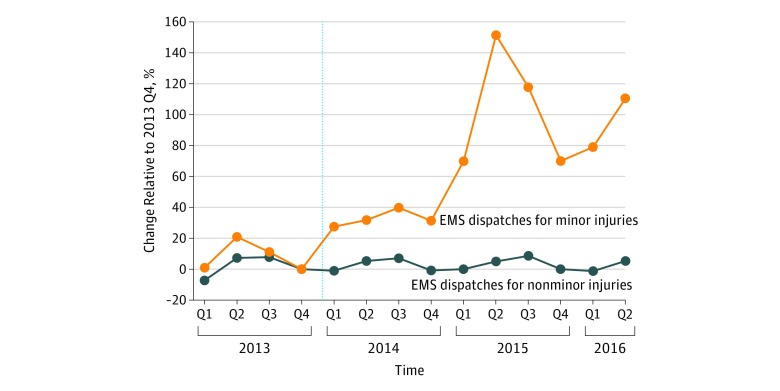
Injury-Related Ambulance Dispatches in New York City, From January 1, 2013, to July 31, 2016 Dispatch volume is reported as a percentage change compared with the fourth quarter (Q) of 2013. In 2013 Q4, there were 1783 minor injury dispatches and 50 684 dispatches for more severe injuries. The dashed line indicates the implementation of the Patient Protection and Affordable Care Act; EMS, emergency medical services.

Figure 2 compares EMS dispatches for minor injuries with dispatches for other types of injuries. By making this comparison, we controlled for any underlying secular trends common to both types of injuries. Again, percentage changes in dispatch volumes compared with the final quarter of 2013 (ie, baseline) are shown. Use of ambulances for minor injuries followed the same general trend as other injuries prior to the implementation of the ACA. Then, in the first quarter of 2014, the 2 trends diverged, with minor injuries becoming relatively more frequent. By the second quarter of 2015, there was a 150% increase in dispatches for minor injuries compared with baseline. Although it is not possible to discern which of these dispatches were medically unnecessary, it is reasonable to speculate that much of this increase was associated with the ACA insulating patients from having to pay the full cost of EMS.

### Difference-in-Differences Regression Estimates

The ordinary least squares estimate of *β_1_* is reported in Table 2. Its magnitude and precision are consistent with the data presented in Figure 1 and Figure 2. The implementation of the ACA was associated with an increased ordinary least squares estimate of 7.71 (95% CI, 1.23-14.19) increase in EMS dispatches for minor vs other types of injuries per dispatch zone per month, or a 37.2% increase compared with the baseline mean (SD) of 20.75 (14.24) dispatches. Because NYC has 31 dispatch zones, this estimate translates to approximately 239 additional minor injury dispatches per month or 2868 additional dispatches per year.

**Table 2.** Changes in Ambulance Dispatches for Minor Injuries in New York City, New York, After the Implementation of the Patient Protection and Affordable Care Act

Variable	Estimate	
	OLS	Negative Binomial
Difference-in-differences estimate (95% CI)	7.71 (1.23-14.19)	0.46 (0.43-0.48)
*P* value	.02^a^	<.001^a^
Change, %^b^	37.2	58.4
Observations, No.	2604	2604

Abbreviation: OLS, ordinary least squares.

^a^ Statistical significance denoted by *P* < .05.

^b^ Using OLS, the percentage change was calculated using the volume of minor injury dispatches in 2013 as the baseline (ie, 7.71/20.75). Using negative binomial regression analysis, the estimated coefficient was converted by exponentiating and subtracting 1 (ie, *e*^0.46^ − 1 = 0.584).

The negative binomial regression estimate is also positive but larger. The implementation of the ACA is associated with an increase in expected minor injury dispatches of 46 log points (95% CI, 0.43-0.48). This represents a 58.4% increase in minor injury dispatches compared with dispatches for other types of injuries (*e^0^*^.46^ − 1 = 0.584).

### Event-Time Estimates

**Figure 3.**
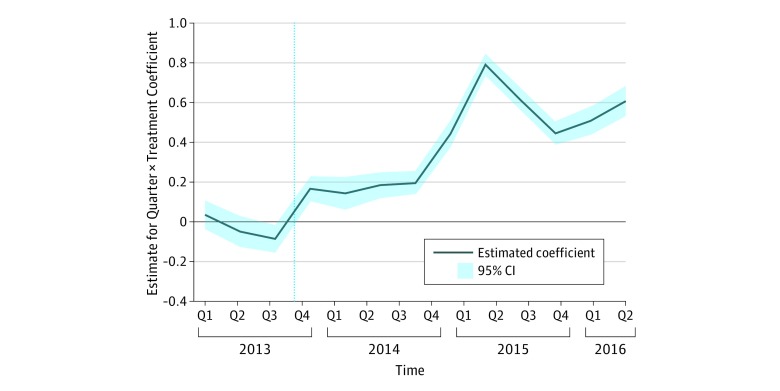
Event-Time Estimates for Minor Injury vs Other Injury Ambulance Dispatches All SEs (used to calculate 95% CIs) are adjusted for clustering at the dispatch-zone level (31 dispatch zones). Observations are at the call type per zone per month level. The outcome is the count of dispatches. The model includes dispatch zone fixed effects and a control for the dispatch zone population in a given year. The first quarter (Q) of 2013 was omitted. The dashed line indicates the implementation of the Patient Protection and Affordable Care Act.

Figure 3 presents the event-time coefficients (ie, the ordinary least squares coefficients of the interactions of *Minor_c_* with the 13 quarter indicators) as well as their 95% CIs, tracing the evolution of minor vs more severe injury ambulance dispatches during the study. Consistent with the parallel-trends assumption, there is no evidence that minor injury dispatches were increasing compared with dispatches for other types of injuries prior to the implementation of the ACA. However, immediately after implementation, minor injury dispatches began to increase. By the fourth quarter of 2015, there was a more than 40% increase in minor injury dispatches compared with dispatches for other types of injuries.

## Discussion

The 2014 study by Taubman et al^21^ provides evidence that the Oregon Medicaid expansion was associated with increased emergency department use. Similarly, Medicaid expansion under the ACA appears to be associated with increased emergency department use; the increase in emergency department use related to injuries appears to have been especially pronounced.^22,23^ A 2017 study^10^ reported evidence that insurance expansions under the ACA are associated with slower ambulance response times.

Our study contributes to the literature by expanding on these various results. The estimates suggest that the implementation of the ACA, which was designed to encourage enrollment and guarantee more generous coverage for enrollees, in NYC was associated with more ambulance dispatches for minor injuries compared with dispatches for other types of injuries.

This positive association is consistent with the argument by Fuchs^24^ that much of the additional emergency care spending associated with insurance expansions is on the so-called flat of the curve. In other words, our findings are consistent with the argument that insurance expansions encourage the use of medical services without leading to better patient outcomes.^25^ Given that the implementation of the ACA may also be associated with ambulance system congestion^10^ and that slower ambulance response times can cost lives,^8,9^ it seems appropriate to ask and ultimately ascertain whether insurance coverage can be expanded without also encouraging the use of EMS for nonemergency cases.

### Strengths and Limitations

One strength of this study is that, because the EIDD file is a census of all ambulance dispatches resulting from 911 calls and because every 911 call classified as a medical emergency results in the dispatch of an ambulance, there were none of the missing data or attrition concerns that often limit the usefulness of observational studies. However, owing to the nonexperimental and observational nature of this study, it was not possible to estimate the causal relationship between the ACA and injury dispatches. The increase in ambulance dispatches for minor injuries compared with those for other types of injuries in NYC after the implementation of the ACA in 2014 could have been associated with patients not having to pay full cost. However, it is also possible that it was associated with another factor that, from the perspective of the researchers, was difficult to observe and measure. For example, it is possible that there was a change in NYC EMS triage practices immediately after the implementation of the ACA. If such a change took place, it was unreported.

External validity represents another important concern. Whether the association of the ACA with EMS dispatches would be similar in other cities is unknown, to our knowledge. The focus of our study was on NYC because of data availability. In 2012, NYC passed an open access law that required that all public data be made available through a single web portal.^14^ As a result, the EIDD file contains detailed information about every ambulance dispatch in NYC, including dispatch time and location information. Most major US cities do not make this information available to the public or researchers, to our knowledge. Until they do, we cannot determine whether the results of our study are generalizable to other ambulance systems and cities.

## Conclusions

The ACA insurance expansion was associated with an increase in ambulance dispatches for minor injuries compared with dispatches for injuries and major injuries in NYC. This association is consistent with the argument that patients will increase their use of unnecessary medical care when they bear a smaller portion of the cost. Such an increase in the use of medical care has important implications for the planning and implementation of health insurance policies. Policy makers must provide appropriate incentives to ensure that patients do not use unnecessary emergency services and overburden delivery systems that, according to many observers,^26,27^ are already stretched to their limits, particularly in low-income areas.^28^

## Notes

Supplement. **eTable.** Most Common Call Types for Each Severity Level
