# Supplementary material for: Association of Ambulance Use in New York City With the Implementation of the Patient Protection and Affordable Care Act
Source: JAMA Netw Open. 2019 Jun 28;2(6):e196419. doi: 10.1001/jamanetworkopen.2019.6419 (PMC6604083; doi:10.1001/jamanetworkopen.2019.6419)
Supplement: Supplement. — eTable. Most Common Call Types for Each Severity Level [file jamanetwopen-2-e196419-s001.pdf]

Supplementary Online Content

Courtemanche C, Friedson AI, Rees DI. Association of ambulance use in New York City with the implementation of the Patient Protection and Affordable Care Act. *JAMA Netw Open*. 2019;2(6):e196419. doi:10.1001/jamanetworkopen.2019.6419

eTable. Most Common Call Types for Each Severity Level

This supplementary material has been provided by the authors to give readers additional information about their work.

© 2019 Courtemanche C et al. *JAMA Network Open.*

eTable. Most Common Call Types for Each Severity Level [a]

Call Severity	Predominant Call Types
1	Cardiac Arrest, Choking
2	Anaphylaxis, Asthma Attach, Cardiac Condition with Difficulty Breathing, Cerebrovascular Accident (Stroke), Difficulty Breathing, Drowning, Jumper Down, Obstetric Complications, Status Epilepticus (Seizure), Multiple Trauma, Unconscious Patient
3	Altered Mental Status, Cardiac Condition, Gunshot, Major Burn, Major Injury, Major Obstetric Complaint, Pedestrian Struck, Stabbing
4	Prescription Drug or Alcohol Abuse, Motor Vehicle Accident, Unknown Medical Situation
5	Abdominal Pain, Injury, Female in Labor
6	Sick Patient
7	Psychiatric Patient, Minor Injury, Minor Illness
8	Ambulance on Standby

[a] Call types are listed if they make up at least 2% of all calls assigned that severity, listed call types make up over 95% of calls in each severity category. The EIDD also includes calls assigned a severity score of 9 for transporting deceased individuals, which makes up less than 0.01% of the data.

© 2019 Courtemanche C et al. *JAMA Network Open.*
